# Effects of exercise amount and intensity versus a combined exercise and lifestyle intervention on metabolic syndrome in adults with prediabetes: a STRRIDE-PD randomized trial

**DOI:** 10.3389/fphys.2023.1199763

**Published:** 2023-07-14

**Authors:** William C. Bennett, Katherine A. Collins, Johanna L. Johnson, Cris A. Slentz, Leslie H. Willis, Connie W. Bales, Kim M. Huffman, William E. Kraus

**Affiliations:** ^1^ Department of Medicine, Duke Molecular Physiology Institute, Duke University School of Medicine, Durham, NC, United States; ^2^ Divison of Geriatrics, Department of Medicine, Durham VA Medical Center, Duke University School of Medicine, Durham, NC, United States; ^3^ Division of Cardiology, Duke University School of Medicine, Durham, NC, United States

**Keywords:** diabetes prevention program, aerobic exercise, diet-and-exercise intervention, lifestyle intervention, diabetes

## Abstract

The purpose of this secondary analysis was to determine what portion of the effects of a Diabetes Prevention Program-like intervention on metabolic syndrome (MetS) could be achieved with exercise alone, as well as to determine the relative importance of exercise intensity and amount to the total exercise effect on MetS. Sedentary, overweight adults with prediabetes were randomly assigned to one of four 6-month interventions: 1) low-amount/moderate-intensity (10 kcal/kg/week at 50% peak $\dot{V}O_{2}$ ); 2) high-amount/moderate-intensity (16 kcal/kg/week at 50% peak $\dot{V}O_{2}$ ); 3) high-amount/vigorous-intensity (16 kcal/kg/week at 75% peak $\dot{V}O_{2}$ ); or 4) diet (7% weight loss) plus low-amount/moderate-intensity (10 kcal/kg/week at 50% peak $\dot{V}O_{2}$ ). The primary outcome of this secondary analysis was change in the MetS z-score. A total of 130 participants had complete data for all five Adult Treatment Panel (ATP) III MetS criteria. The diet-and-exercise group statistically outperformed the MetS z-score and the ATP III score compared to the exercise alone group. Aerobic exercise alone achieved 24%–50% of the total effect of the combined diet-and-exercise intervention on the MetS score. Low-amount moderate-intensity exercise quantitatively performed equal to or better than the interventions of high-amount moderate-intensity or high-amount vigorous-intensity exercise in improving the MetS score. The combined diet-and-exercise intervention remains more efficacious in improving the MetS z-score. However, all three exercise interventions alone showed improvements in the MetS z-score, suggesting that a modest amount of moderate-intensity exercise is all that is required to achieve approximately half the effect of a diet-and-exercise intervention on the MetS.

**Clinical Trial Registration:** clinicaltrials.gov, identifier NCT00962962.

## Introduction

Individuals diagnosed with metabolic syndrome (MetS) are at increased risk for developing both type 2 diabetes mellitus and cardiovascular disease (Grundy et al., 2005; Mottillo et al., 2010; Tune et al., 2017); however, aerobic exercise can be used as a preventative treatment to combat these diseases (Colberg et al., 2010; Kirwan et al., 2017; Physical Activity Guidelines Advisory Committee, 2018; Adams and Linke, 2019; Tian and Meng, 2019; Li et al., 2020). The Diabetes Prevention Program (DPP) implemented a diet aimed at 7% weight loss, combined with aerobic exercise, and found it to be superior to sole pharmacologic therapy for progression to diabetes for individuals who have prediabetes (Group, 2002a; Knowler et al., 2002). Although the benefits of both diet and exercise combined on MetS are well known, the contribution of aerobic exercise alone to improve MetS remains relatively unknown.

The National Cholesterol Education Program (NCEP) Adult Treatment Panel III (ATP III) clinically defines MetS as having three or more of the following five criteria: 1) abdominal obesity, 2) increased triglyceride levels, 3) low HDL-cholesterol, 4) increased blood pressure, and 5) increased fasting glucose levels (Grundy et al., 2005). Finding the appropriate amount and intensity exercise prescription for improvements in MetS is clinically important to individuals who are unable or unwilling to adhere to a dietary intervention. These findings are also important for clinicians who find it difficult to initiate two lifestyle interventions (diet and exercise) for their patients during limited face-to-face time in their practices. This approach needs to be explored in MetS.

Thus, the purpose of this secondary analysis of the Studies of a Targeted Risk Reduction Interventions through Defined Exercise in Pre-Diabetes (STRRIDE-PD) was to determine what portion of the effects of a DPP-like intervention on MetS could be achieved with exercise alone, as well as to determine the relative importance of exercise intensity and amount to the total exercise effect. Furthermore, some aspects of this analysis were motivated by our previous findings in STRRIDE I. Participants were randomly assigned to 6-month control, low-amount/moderate-intensity, low-amount/vigorous-intensity, or high-amount/vigorous-intensity groups. The low-amount/moderate-intensity group significantly improved the MetS z-score relative to the control group. However, the same amount of exercise at vigorous intensity did not significantly improve the MetS z-score more than the control group, suggesting lower-intensity exercise may be more effective in improving MetS (Johnson et al., 2007).

## Materials and methods

### Study design

In the STRRIDE-PD randomized trial (NCT00962962), participants were recruited continuously between 2009 and 2012 from Durham, NC, United States, and the surrounding area. Participants completed anthropometric, blood pressure, and blood collection assessments prior to and following a 6-month supervised exercise intervention (Slentz et al., 2016). Randomization was performed using a standard computer-based random number generator using a randomized design, blocked by gender and race. The patients provided their verbal and written informed consent. The protocol was approved by the Duke University Institutional Review Board.

### Participants

Potential participants (*n* = 3,052) responded to local advertisements and were phone-screened. Of these, 288 met the inclusion criteria and were enrolled in the study. The inclusion criteria were as follows: age 45–75 years, body mass index (BMI) 25–35 kg/m^2^, resting blood pressure <160/90 mmHg, fasting plasma glucose ≥95 to <126 mg/dL (readings taken on 2 separate days, both being ≥95 mg/dL and the first being <126 mg/dL), and low-density lipoprotein (LDL) cholesterol <190. The exclusion criteria included smoking, diabetes, uncontrolled hypertension, musculoskeletal disorders, and cardiovascular disease. Participants were free from medications that would influence either formulation of MetS used in this study. A complete description of the STRRIDE-PD study design has been described elsewhere (Slentz et al., 2016). Participants were compensated for participation in the STRRIDE-PD trial.

### Interventions

Participants were randomly assigned to one of four 6-month intervention groups: 1) low-amount/moderate-intensity exercise—energy expenditure of 10 kcal per kg of body weight per week (KKW) at 50% $\dot{V}O_{2}$ reserve; 2) high-amount/moderate-intensity exercise—16 KKW at 50% $\dot{V}O_{2}$ reserve; 3) high-amount/vigorous-intensity exercise—16 KKW at 75% $\dot{V}O_{2}$ reserve; and 4) clinical lifestyle intervention (diet + exercise)—10 KKW at 50% $\dot{V}O_{2}$ reserve plus a diet designed to reduce body weight by 7% over 6 months (Slentz et al., 2016). The $\dot{V}O_{2}$ peak was determined by a graded maximal treadmill test which started at 3 mph with 0% grade and then increased speed and/or grade such that the metabolic demand increased at approximately 3.5 mL/kg/min (1 MET) per stage until volitional exhaustion. According to the exercise group prescription, the $\dot{V}O_{2}$ reserve (50% or 75%) was determined as 0.50 or 0.75 × ( $\dot{V}O_{2}$ peak − resting $\dot{V}O_{2}$ ) + resting $\dot{V}O_{2}$ . The weekly exercise energy expenditure (KKW) was determined based on the percentage of the $\dot{V}O_{2}$ reserve only, without including the resting $\dot{V}O_{2}$ , as this is the most accurate way to compare exercise energy expenditure in groups exercising at different intensities (Howley, 2001). The heart rate at any given intensity was determined by a submaximal, progressive exercise test with gas exchange. The exercise modality used for the exercise intervention consisted primarily of treadmills, but also included elliptical trainers, rowing, and cycle ergometers. Exercise frequency was determined by the participant; however, subjects were encouraged to adhere to a weekly schedule that required no more than 60 min to complete a single exercise session. The participants were asked to exercise under direct supervision at least 2 days per week to allow the study staff to download files from heart rate monitors and discuss training progress throughout the intervention period. Garmin heart rate monitors (Olathe, KS, United States) with stored exercise files enabled participants to exercise at the fitness center without direct supervision as required during the week while still allowing the study staff to monitor protocol compliance.

The total weekly exercise amount was gradually increased over a 10-week ramp period, while the prescribed exercise intensity was maintained. As fitness increased, participants needed to work harder to achieve their target heart rate, which required a reduction in weekly exercise time to maintain constant weekly energy expenditure. Target heart rates were confirmed by submaximal oxygen consumption testing at the midpoint of the exercise program. The maximal exercise duration was capped at 6 h per week to avoid a disproportionate time burden and potentially reduced protocol compliance among participants with low exercise capacity—as those with a lower VO_2_ peak required more minutes of exercise per week to expend the same total energy per week.

Participants randomized to the diet-and-exercise group received an intervention modeled after the DPP (Group, 2002b), designed to achieve a 7% weight reduction via energy-intake restriction and low-fat diet and exercise over a 6 month intervention period. The total energy expenditure was calculated based on weight, height, gender, age, and activity level using equations published by the Institute of Medicine (2005). The total energy expenditure was then adjusted downward to achieve approximately 1–2 pounds of weight loss (average of 0.6 kg) per week, which, in general, resulted in a reduction of approximately 500 kcal per day. Following four initial counseling sessions, the participants attended 12 bi-weekly intensive group sessions adapted from the DPP manual.

### Anthropometrics and blood pressure measurements

Body height and weight were measured in light clothing and without shoes to the nearest 0.1 kg on a digital scale (Scale 5005; Scale-Tronix Inc., Wheaton, IL). Height was measured to the nearest 0.5 cm. BMI was calculated as weight (kilograms) divided by height (meters) squared. Waist circumference was measured horizontally at the minimal waist, the narrowest portion of the torso, between the umbilicus and xiphoid process (Willis et al., 2007). Two blood pressure readings were obtained after a 60-min rest, and the average of the two readings was used for the present analysis. There was a 60-s rest period between the first and second blood pressure readings.

### Plasma lipids and fasting glucose levels

The participants were asked to eat their normal diet the evening before each blood collection, which were prior to and following the intervention period. Plasma glucose levels, total cholesterol, HDL-cholesterol, and triglycerides levels were measured using a Beckman Coulter DxC600 clinical analyzer (Brea, CA, United States).

### Metabolic syndrome z-score

As previously described in earlier STRRIDE studies, the MetS z-score used in the present study was a continuous score based on the five MetS variables (Johnson et al., 2007; Bateman et al., 2011). A modified z-score was calculated for each variable using individual participant data, the ATP III criteria, and standard deviations derived from participants who had complete pre- and post-intervention MetS data from the STRRIDE-PD cohort at baseline (*n* = 130). Gender-specific MetS z-score equations were used to account for variations in the ATP III criteria for men and women. HDL-cholesterol (HDL-C) and waist circumference were determined separately for men and women given there are consistent differences in mean values (standard deviation). The equations used to calculate the MetS z-score were as follows:

Men z-score = [(40 − HDL)/10.0] + [(TG −150)/58.8] + [(fasting blood glucose − 100)/9.6] + [(waist circumference − 102)/6.9] + [(mean arterial pressure − 100)/9.5].

Women z-score = [(50 − HDL)/11.9] + [(TG − 150)/58.8] + [(fasting blood glucose − 100)/9.6] + [(waist circumference − 88)/7.6] + [(mean arterial pressure − 100/9.5].

A MetS risk factor score using the ATP III guidelines was also determined for each participant as a sum (0–5) of the number of ATP III criteria met before and after the exercise intervention.

### Statistical analyses

Data were analyzed using JMP^®^ 15.1 (SAS Institute, Cary, NC, United States). Prior to data analysis, all assumptions for each statistical test were assessed. To assess for significant within-group changes, we conducted two-tailed, paired *t*-tests comparing post-minus pre-intervention values. A *p*-value of <0.05 was considered significant. To determine the proportion each exercise-only group achieved compared to the diet-and-exercise group for each figure, we used the following calculation:

$$\frac{\left|Exercise\;only\;group\;change\;score\right|}{\left|Diet\;and\;exercise\;group\;change\;score\right|}*100.$$

To determine whether significant between-group differences existed, analysis of covariance (ANCOVA), with baseline values used as covariates, was conducted. If an ANCOVA *p*-value was <0.10, *post hoc* testing was conducted using the Tukey–Kramer adjustment for multiple comparisons. *Post hoc* testing only assessed between-group differences among the exercise-only groups and the diet-and-exercise group. Because the variables in the present article were not the primary outcome variables for the STRRIDE-PD trial, there were no *a priori* power calculations. The analyses presented were performed as “per protocol.”

## Results

**FIGURE 1:**
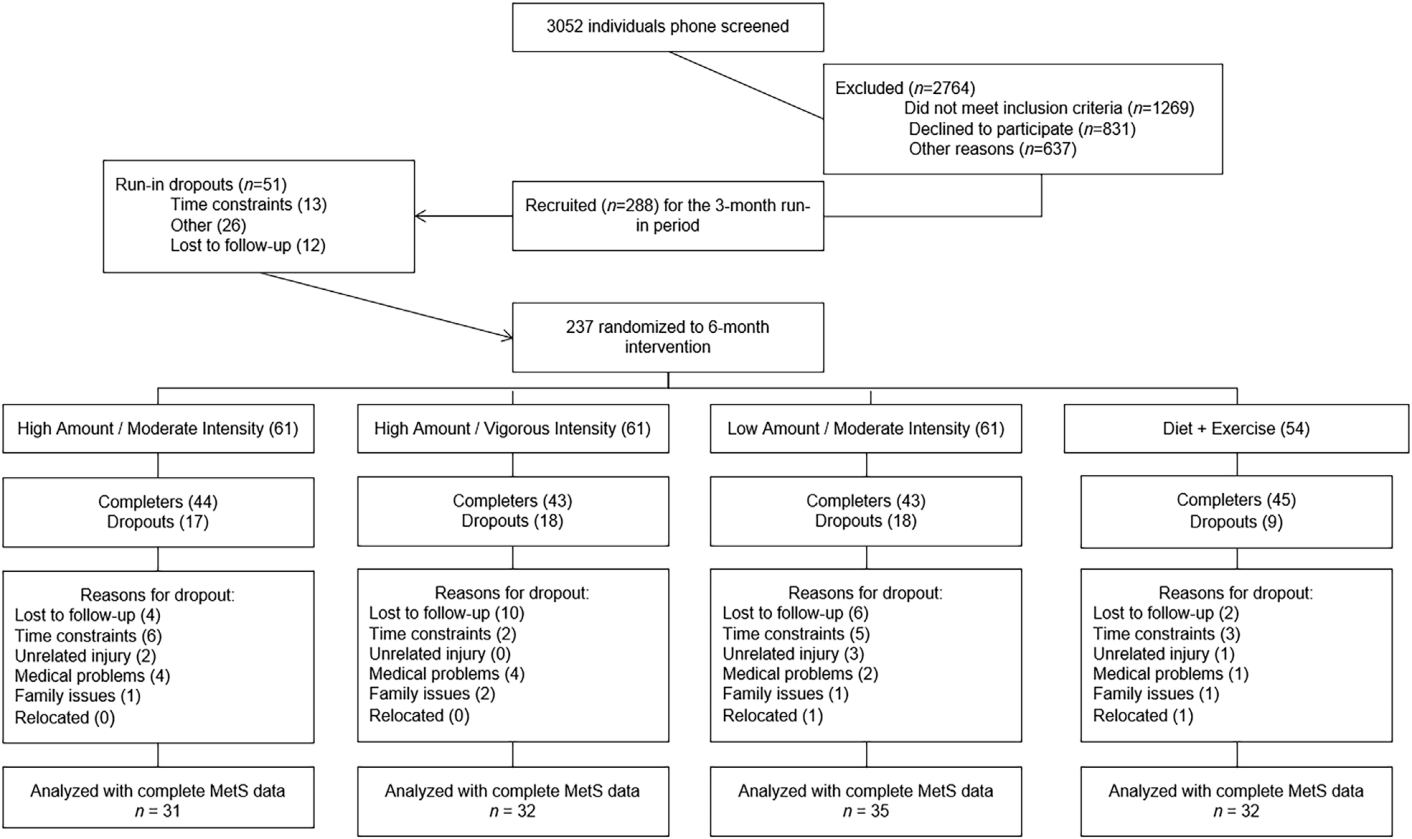
Flowchart of screening, randomization, inclusion, and exclusion.

Of the 237 participants randomized, 130 had both pre-intervention and post-intervention data available for the five variables constituting MetS. Figure 1 describes the flow of participants from recruitment to post-intervention testing. Baseline characteristics and exercise prescriptions are given for each group in Table 1. Participants were on average 59.7 ± 7.4 years, with obesity (BMI: 30.4 ± 2.6 kg/m^2^), predominately female (58%), and white (79%). Exercise adherence was similar across groups; however, the high-amount/moderate-intensity group averaged 79 more minutes of aerobic exercise per week, which required the completion of a fourth exercise session each week for most participants.

**TABLE 1:** Demographic and baseline characteristics.

Variable	Total (n = 130)	Low amount/moderate intensity (n = 35)	High amount/moderate intensity (n = 31)	High amount/vigorous intensity (n = 32)	Diet + exercise (n = 32)
Age, years	59.7 (7.4)	57.9 (7.5)	61.0 (6.9)	61.0 (7.1)	59.2 (7.9)
Weight, kg	86.6 (12.6)	88.3 (12.4)	85.6 (12.2)	85.6 (11.0)	86.8 (14.8)
BMI, kg/m^2^	30.5 (2.8)	30.7 (2.7)	30.1 (2.8)	30.6 (2.7)	30.6 (3.0)
Race, *n*					
Caucasian	103	24	26	26	27
African American/Black people	22	5	5	4	8
Others	5	3	0	2	0
Sex, *n*					
Female	75	21	18	18	18
Male	55	14	13	14	14
Exercise prescription					
$Intensity,\%\;peak\;\dot{V}O_{2}$		40–55	40–55	65–80	40–55
Amount^a^, kcal x kg^−1^ x wk^−1^		10	16	16	10
Time, min/wk		178.4 (35.6)	286.4 (51.9)	193.5 (38.1)	180.0 (32.9)
Adherence, %		87.4 (20.9)	84.4 (13.7)	88.5 (14.1)	86.9 (14.6)
Actual time, min/wk^b^		158.3 (40.1)	238.6 (54.2)	166.0 (37.6)	156.0 (38.5)
Actual frequency, sessions/wk		3.1 (0.7)	4.0 (0.8)	3.2 (0.6)	3.1 (0.7)

Values are shown as the mean (SD). There were no significant baseline differences across groups. BMI, body mass index.

^a^ Prescription amount (10 and 16 kcal x kg^−1^ x wk^−1^) approximately calorically equivalent to 9 and 14 miles of walking/jogging per week.

^b^ Actual time (min/wk) = time x adherence.

Baseline and change scores for the MetS z-score, ATP III score, and the five MetS criteria are presented for each group in Table 2. Following the intervention, the low-amount/moderate-intensity (−0.9 ± 1.8; *p* = 0.005), high-amount/vigorous-intensity (−1.0 ± 1.9; *p* = 0.008), and diet-and-exercise (−2.4 ± 2.0; *p* < 0.0001) groups significantly improved (decreased) MetS z-scores, with the high-amount/moderate-intensity group trending toward significance (−0.6 ± 1.7; *p* = 0.07). Low-amount/moderate-intensity (−0.3 ± 0.8; *p* = 0.02) and diet-and-exercise (−0.6 ± 0.9; *p* = 0.0007) groups significantly decreased ATP III score, with no significant change in the ATP III score for the two high-amount intervention groups (high-amount/moderate-intensity change score = −0.2 ± 1.0; high-amount/vigorous-intensity change score = −0.2 ± 1.1). The diet-and-exercise group showed the most robust improvements for change in the five MetS criteria significantly decreasing waist circumference (−5.0 ± 4.9 cm; *p* < 0.0001), triglyceride levels (−25.5 ± 32.0 mg/dL; *p* < 0.0001), mean arterial pressure (−4.5 ± 8.8 mmHg; *p* = 0.007), and fasting blood glucose levels (−4.8 ± 5.6 mg/dL; *p* < .0001), with a trending change in HDL-cholesterol post-intervention (1.9 ± 5.7 mg/dL; *p* = 0.06). Among the exercise-only groups, waist circumference significantly decreased for both high-amount/moderate-intensity (−2.5 ± 4.2 cm; *p* = 0.003) and high-amount/vigorous-intensity (−2.1 ± 3.4 cm; *p* = 0.001) groups. HDL-cholesterol levels significantly increased within the low-amount/moderate-intensity (2.0 ± 4.9 mg/dL; *p* = 0.02) and high-amount/vigorous-intensity (1.7 ± 3.5 mg/dL; *p* = 0.01) groups. Fasting blood glucose levels significantly decreased in the high-amount/moderate-intensity group (−2.7 ± 6.8 mg/dL; *p* = 0.04). The low-amount/moderate-intensity group experienced a significant decrease in mean arterial pressure (−2.9 ± 8.4 mmHg; *p* = 0.05). No significant within-group changes in triglyceride levels were found for the exercise-only groups.

**TABLE 2:** Baseline values and change scores for metabolic syndrome variables and scores across intervention groups.

Variable	Low amount/moderate intensity		High amount/moderate intensity		High amount/vigorous intensity		Diet + exercise	
	Baseline	Change	Baseline	Change	Baseline	Change	Baseline	Change
Body mass, kg	88.3 (12.4)	−1.1 (3.1)*	85.6 (12.2)	−2.1 (2.8)**	85.6 (11.0)	−1.7 (2.2)**	86.8 (14.8)	−6.4 (5.2)**
BMI, kg/m^2^	30.7 (2.6)	−0.5 (1.2)*	30.1 (2.8)	−0.8 (1.0)**	30.6 (2.7)	−0.6 (0.8)**	30.6 (3.0)	−2.1 (1.7)**
Waist circumference, cm	108.0 (6.6)	−0.9 (4.4)	108.0 (7.0)	−2.5 (4.2)**	107.0 (8.2)	−2.1 (3.4)**	108.0 (8.4)	−5.0 (4.9)**
HDL-cholesterol, mg/dL	42.9 (11.9)	2.0 (4.9)*	44.0 (13.4)	0.5 (4.7)	45.0 (12.1)	1.7 (3.5)*	43.1 (10.8)	1.9 (5.7)^†^
Triglyceride levels, mg/dL	123.0 (55.0)	−10.7 (39.0)*	134.0 (64.0)	−1.7 (41.0)	119.0 (59.0)	−12.8 (45.0)	101.0 (37.0)	−25.5 (32.0)**
Fasting glucose levels, mg/dL	108.0 (11.7)	−0.03 (6.0)	106.0 (9.3)	−2.7 (6.8)*	104.0 (9.0)	0.2 (7.1)	104.0 (7.9)	−4.8 (5.6)**
MAP, mmHg	93.5 (9.2)	−2.9 (8.4)*	91.5 (8.3)	0.4 (8.2)	91.8 (10.4)	−2.4 (8.7)	91.3 (10.4)	−4.5 (8.8)**
SBP, mmHg	126.0 (13.3)	−4.5 (13.1)*	127.0 (16.0)	−0.3 (13.1)	126.0 (11.2)	−1.8 (12.3)	123.0 (12.4)	−4.0 (12.5)^†^
DBP, mmHg	77.5 (8.7)	−2.1 (7.4)	74.0 (6.8)	0.8 (7.5)	74.5 (11.7)	−2.1 (8.3)	75.5 (10.6)	−4.7 (9.1)**
Relative VO_2_, mL/kg/min	25.0 (5.7)	1.3 (2.6)**	24.4 (4.9)	1.5 (2.3)**	24.3 (5.4)	2.5 (2.5)**	24.5 (4.4)	2.5 (2.7)**
MetS z-score	1.4 (2.6)	−0.9 (1.8)**	1.0 (2.1)	−0.6 (1.7)^†^	0.5 (3.6)	−1.0 (1.9)**	0.5 (2.5)	−2.4 (2.0)**
ATP III Score	3.3 (0.9)	−0.3 (0.8)*	3.0 (0.9)	−0.2 (1.0)	2.7 (1.3)	−0.2 (1.1)	2.8 (1.1)	−0.6 (0.9)**

Values are shown as the mean (SD). There were no significant baseline differences across groups. BMI, body mass index; MAP, mean arterial pressure; SBP, systolic blood pressure; DBP, diastolic blood pressure. The *p*-value represents significance within group changes; ^†^ *p* < 0.1; *p < 0.5; and **p < 0.01.

**FIGURE 2:**
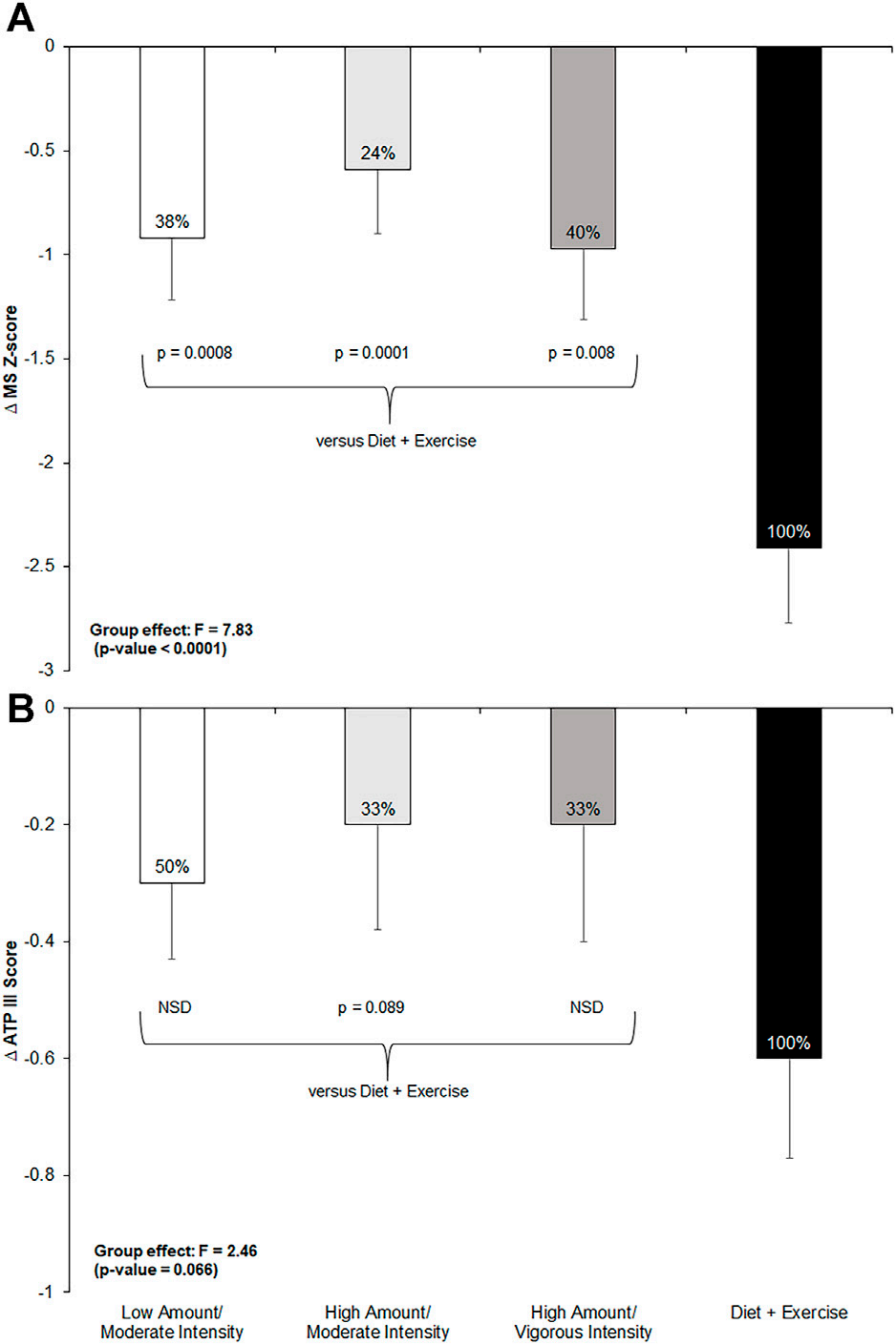
Change in metabolic syndrome (MetS) z-score and ATP III score from pre- to post-intervention across all groups and post-hoc comparisons for each measure if *p*-value <0.1 from ANCOVA. NSD = non-significant difference versus diet-and-exercise group. Low-amount/moderate-intensity *n* = 35; high-amount/moderate-intensity *n* = 31; high-amount/vigorous-intensity *n* = 32; diet-and-exercise *n* = 32. **(A)**: change in MetS z-score by intervention group. **(B)**: change in ATP III score by intervention group.

The exercise-only groups achieved 24%–50% of the total effect of the combined diet-and-exercise intervention on the MetS z-score and ATP III score (Figure 2). There were significant between-group differences found for changes in the MetS z-score among intervention groups (F = 7.83; *p* < 0.0001). *Post hoc* analyses revealed significant between-group differences among all three exercise-only groups (low-amount/moderate-intensity: *p* = 0.0008; high-amount/moderate-intensity: *p* = 0.0001; and high-amount/vigorous-intensity: *p* = 0.008) compared to the diet-and-exercise group for the MetS z-score. Moreover, there was an impressionable between-group effect found for changes in ATP III score among the intervention groups (F = 2.46; *p* = 0.07). However, *post hoc* testing only found a trending difference between the diet-and-exercise and high-amount/moderate-intensity groups for ATP III score (*p* = 0.09).

**FIGURE 3:**
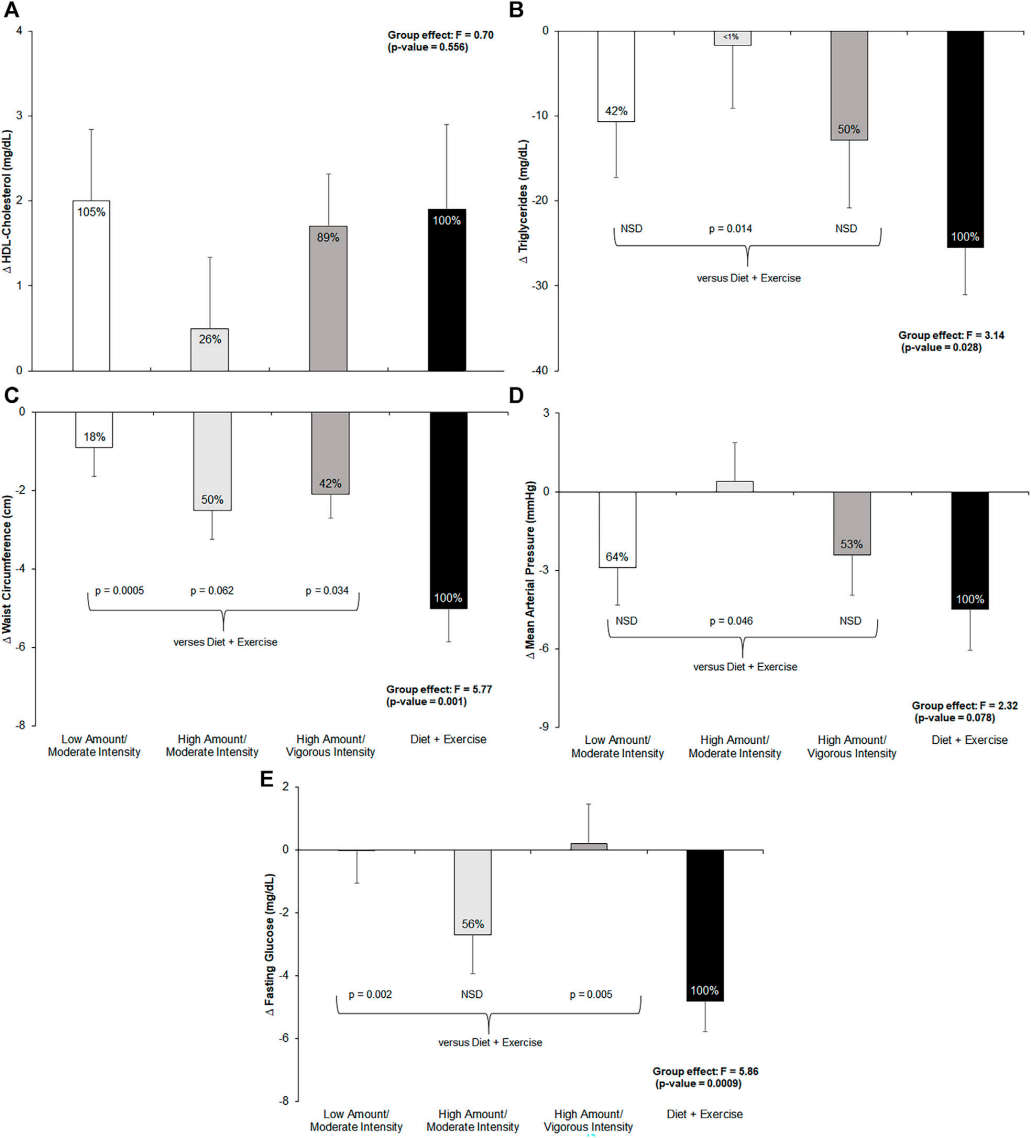
Change in metabolic syndrome (MetS) components from pre- to post-intervention across all groups and post-hoc comparisons for each measure if *p*-value <0.1 from ANCOVA. NSD = non-significant difference versus diet-and-exercise group. Low-amount/moderate-intensity *n* = 35; high-amount/moderate-intensity *n* = 31; high-amount/vigorous-intensity *n* = 32; diet-and-exercise *n* = 32. **(A)**: change in HDL-cholesterol (mg/dL) by intervention group. **(B)**: change in triglycerides (mg/dL) by intervention group. **(C)**: change in waist circumference (cm) by intervention group. **(D)**: change in mean arterial pressure (mmHg) by intervention group. **(E)**: change in fasting glucose (mg/dL) by intervention group.

Figure 3 shows the pre- to post-intervention change in each of the five MetS criteria across all four intervention groups, the total effect the exercise groups achieved compared to the diet-and-exercise intervention, and *post hoc* comparisons. The exercise-only groups achieved 18%–50% of the total effect of the diet-and-exercise intervention, with a significant between-group effect found (F = 5.77; *p* = 0.001). *Post hoc* comparisons revealed a significant difference among the low-amount/moderate-intensity (*p* = 0.0005) and high-amount/vigorous-intensity (*p* = 0.03) groups and the diet-and-exercise intervention, with a trending difference between the high-amount/moderate-intensity (*p* = 0.06) group and the diet-and-exercise group. The low-amount/moderate-intensity group surpassed the total effect of the diet-and-exercise intervention for changes in HDL-cholesterol, with all three exercise-only groups achieving 26%–105% of the effect; however, no significant between-group differences were found among the exercise-only groups and the diet-and-exercise intervention. The high-amount/moderate-intensity group achieved over half (56%) of the diet-and-exercise effect, with the remaining exercise groups experiencing nearly no effect. A significant between-group effect was found for the change in fasting glucose levels across the intervention (F = 5.86; *p* = 0.0009). *Post hoc* analyses revealed significant differences between the diet-and-exercise group and both the low-amount/moderate-intensity (*p* = 0.002) and high-amount/vigorous-intensity (*p* = 0.005) groups. The low-amount/moderate-intensity and high-amount/vigorous-intensity groups achieved 64% and 53% of the diet-and-exercise effect, respectively, for mean arterial pressure. An impressionable between-group effect was observed for changes in mean arterial pressure across the intervention (F = 2.32; *p* = 0.08). *Post hoc* analyses revealed a nearly significant difference between the diet-and-exercise group and high-amount/moderate-intensity group (*p* = 0.05). The high-amount/vigorous-intensity and low-amount/moderate-intensity groups achieved 50% and 42% of the effect of the diet-and-exercise group, respectively, on triglyceride levels. A significant between-group effect was found for changes in triglyceride levels across the intervention (F = 3.14; *p* = 0.03). *Post hoc* testing revealed a significant difference between the diet-and-exercise group and the high-amount/moderate-intensity group (*p* = 0.01).

**FIGURE 4:**
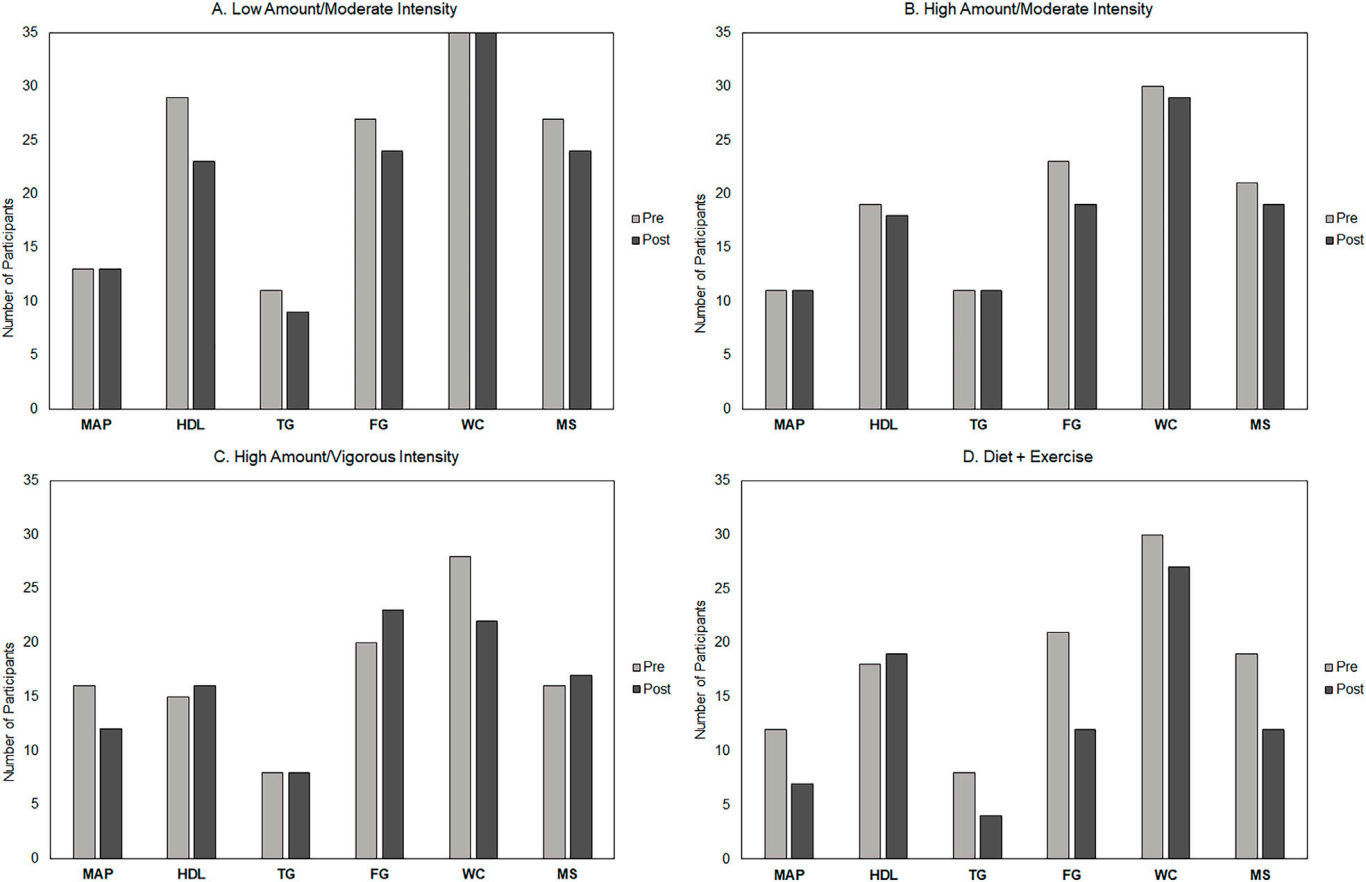
Pre- and post-intervention distribution of metabolic syndrome (MetS) components and number of participants with MetS across groups. Low-amount/moderate-intensity *n* = 35; high-amount/moderate-intensity *n* = 31; high-amount/vigorous-intensity *n* = 32; and diet-and-exercise *n* = 32. MAP, mean arterial pressure; HDL-C, high-density lipoprotein cholesterol; TG, triglycerides; FG, fasting glucose; WC, minimal waist circumference; MS, metabolic syndrome.

Figure 4 shows the pre- and post-intervention distribution of each MetS parameter and number of participants with MetS across intervention groups. The diet-and-exercise group demonstrated the greatest decrease in the prevalence of MetS from baseline (*n* = 19; 59%) to post-intervention (*n* = 12; 38%). For the exercise-only groups, the two moderate-intensity groups showed similar decreases in the prevalence of MetS—high-amount/moderate-intensity [baseline *n* = 21 (68%); post-intervention n = 19 (61%)] and low-amount/moderate-intensity [baseline *n* = 27 (77%); post-intervention *n* = 24 (69%)]. Interestingly, the high-amount/vigorous-intensity group showed an increase in the MetS prevalence [baseline *n* = 16 (50%); post-intervention *n* = 17 (53%)].

## Discussion

To the best of our knowledge, this secondary analysis is the first to determine what percentage of the “gold standard” diet-and-exercise effect on MetS and the five associated risk factors can be achieved with aerobic exercise alone. In addition, we sought to determine the independent effects of exercise intensity and amount on these same parameters among individuals at risk for diabetes.

The most definitive observation of this secondary analysis was that a combination of diet and exercise was more efficacious than exercise alone at improving MetS in both ATP III score and MetS z-score. Individual risk factors of MetS—waist circumference, triglyceride levels, fasting glucose levels, and mean arterial pressure—significantly decreased in the diet-and-exercise group. HDL-cholesterol showed improvements but not to the point of statistical significance. Although the exercise-only groups showed improvements in MetS, the effects were not as robust or widespread as for the diet-and-exercise group nor were there significant differences among the various exercise groups matched in various combinations of intensity and amount. Exercise-only groups achieved 24%–50% of the total effect of the combined diet and exercise intervention on the MetS z-score, where a low-amount moderate-intensity exercise quantitatively performed equal to or better on this measure than the high-amount vigorous-intensity exercise and high-amount moderate-intensity exercise.

The effects of exercise alone on the individual components of MetS revealed a different story. The effects of exercise alone on HDL-cholesterol were statistically indistinguishable from those of diet-and-exercise. Furthermore, the effect of moderate-intensity exercise of either amount was quantitatively indistinguishable from that of diet-and-exercise, and moderate-intensity exercise was quantitatively better than vigorous-intensity exercise. However, this pattern was not replicated for the effects of exercise alone on triglyceride levels, where moderate-intensity exercise of either amount did not statistically outperform vigorous-intensity exercise, with vigorous-intensity exercise achieving 50% of the effect found in the diet-and-exercise group.

Interestingly, a high amount of exercise at either intensity achieved approximately 50% of the effect of the combined diet and exercise intervention on reductions in waist circumference among individuals with prediabetes. Similarly, exercise alone achieved up to 64% of the effect of the combined diet and exercise intervention on mean resting blood pressure, where a low-amount moderate-intensity exercise performed similar to a high-amount vigorous-intensity exercise. We observed this pattern previously in our prior STRRIDE I trial (Johnson et al., 2007). Finally, a high-amount of moderate-intensity exercise alone can reduce fasting glucose levels up to 56% of the effect of diet-and-exercise intervention. In general, the sum of these findings has important implications for individuals who are unable or unwilling to adhere to a dietary intervention, as well as for clinicians with limited face-to-face time in their practices. Under these circumstances, encouraging individuals to implement exercise alone into their daily routine may improve the uptake of exercise participation, in addition to moderately improving MetS. However, when targeting a beneficial response among the individual components of MetS, an individualized exercise prescription approach may be best.

The present analysis points to the power of moderate-intensity exercise (brisk walking) when addressing the underlying pathophysiology associated with MetS. Moderate-intensity exercise alone compares favorably to a diet-and-exercise intervention, achieving close to 50% of the benefit found from the more intensive diet-and-exercise program on all five components of MetS—HDL-cholesterol, triglyceride levels, waist circumference, mean blood pressure, and fasting plasma glucose levels. This finding confirms that of what was observed in the STRRIDE I trial, moderate-intensity exercise significantly improves insulin sensitivity compared to vigorous-intensity exercise of the same amount (Houmard et al., 2004; Slentz et al., 2009). Similarly, moderate-intensity exercise significantly improved the MetS z-score (Johnson et al., 2007). However, there are two major advances over our previous work in this analysis. First, in the present study, all individuals were prediabetic and therefore at a greater risk for diabetes than those in our previous STRRIDE trials, where approximately 33% met prediabetes criteria. Second, in STRRIDE I, comparisons of exercise-alone groups were made with an inactive control group. Here, exercise alone was compared to a gold standard lifestyle intervention (DPP) being implemented clinically throughout the US. Of note, although the diet-and-exercise group was modeled after the DPP, findings from this analysis have broader implications. Given the substantial improvements in MetS and the five parameters of MetS observed in the diet-and-exercise group, our findings can be generalized to interventions and programs aiming to prevent or improve dyslipidemia, hypertension, and obesity.

The effects of exercise on MetS and the associated risk factors have been demonstrated in previous studies (Katzmarzyk et al., 2003; Johnson et al., 2007; Kemmler et al., 2009; Stensvold et al., 2010; Bateman et al., 2011; Morales-Palomo et al., 2019). However, the extent to which exercise alone can achieve the effects of a combined diet-and-exercise program on MetS and its various components is less understood. The Oslo Diet and Exercise Study was a 1-year randomized controlled trial examining the alone and combined effects of a diet and exercise intervention on MetS among middle-aged men. The study found that the percent of participants with MetS was reduced by 67.4% in the diet-and-exercise combined group, 23.5% in the exercise-only group, and 35.3% in the diet-only group (Anderssen et al., 2007). Similarly, we found that, following the intervention, the diet-and-exercise group showed the greatest decrease in the prevalence of MetS, with a 37% decrease. However, our moderate-intensity exercise-only groups only achieved a 10%–11% decrease in prevalence. The difference in effects may be due to the relative durations of the intervention in the two studies—Oslo being 1 year and the present study being half as long. Another large randomized controlled trial, the Diet and Exercise for Elevated Risk (DEER) trial, determined the effects of a 1-year intervention of a low-fat diet plus exercise, low-fat diet alone, exercise alone, or sedentary control group on the MetS z-score. Using the same constructed MetS z-score calculation as the current analysis, the diet-and-exercise group showed a greater improvement than the control group. However, when using change in body fat as a covariate, those differences were no longer present (Camhi et al., 2010). Similarly, the diet-and-exercise group had the greatest improvement in the MetS z-score compared to the exercise-only intervention groups.

The major strengths of this study are as follows: 1) direct comparison of a diet-and-exercise intervention with exercise alone; 2) the diet-and-exercise intervention was modeled after the DPP with an exercise prescription of 10 KKW at 50% VO_2_ reserve and a diet intervention aimed to reduce body weight by 7% over 6 months; and 3) direct verification of time and intensity for nearly all exercise training sessions. However, this study has some minor limitations. First, the high-amount/moderate-intensity group was capped at 6 h per week to avoid participant burden. Second, the diet part of the combined diet-and-exercise group involved more one-on-one and small group interactions, resulting in the least number of dropouts of all groups. This difference in retention observed for the combined diet-and-exercise group may have confounded the conclusion. Third, each individual likely responded differently to the same exercise and/or diet prescription; thus, future research should investigate individual heterogeneity and responsiveness across MetS and its components when developing targeted intervention approaches. Fourth, given that this is a secondary analysis, power was conducted *a priori* based on the primary outcome for the STRRIDE-PD trial. Lastly, approximately half of the participants did not have MetS prior to the start of the intervention, potentially limiting our ability to detect smaller differences. Additionally, future research should investigate the influence changes in sedentary behavior and light-intensity physical activity may have on MetS and its components.

In summary, exercise alone—varying in intensity and amount—achieves 24%–50% of the benefit found in the combined diet-and-exercise intervention on the MetS z-score. A low amount of moderate-intensity exercise quantitatively performs equal to or better on changes in the MetS z-score compared to a greater amount of vigorous- or moderate-intensity exercise, suggesting that lesser amounts of moderate-intensity exercise can effectively achieve half of the benefit of a diet-and-exercise intensive lifestyle intervention on MetS. However, when targeting individual components of MetS, individualized exercise programs—varying in intensity and amount—may be best suited.

## Data Availability

The raw data supporting the conclusion of this article will be made available by the authors, without undue reservation, upon reasonable request.
